# Lung Involvement in Systemic Juvenile Idiopathic Arthritis: A Narrative Review

**DOI:** 10.3390/diagnostics12123095

**Published:** 2022-12-08

**Authors:** Duilio Petrongari, Paola Di Filippo, Francesco Misticoni, Giulia Basile, Sabrina Di Pillo, Francesco Chiarelli, Marina Attanasi

**Affiliations:** Department of Pediatrics, University of Chieti, 66100 Chieti, Italy

**Keywords:** sJIA-LD, systemic juvenile idiopathic arthritis and lung disease, DRESS, tocilizumab, anti-IL-1, anti-IL-6, cytokine plasticity hypothesis, digital clubbing

## Abstract

Systemic juvenile idiopathic arthritis associated with lung disorders (sJIA-LD) is a subtype of sJIA characterized by the presence of chronic life-threatening pulmonary disorders, such as pulmonary hypertension, interstitial lung disease, pulmonary alveolar proteinosis and/or endogenous lipoid pneumonia, which were exceptionally rare before 2013. Clinically, these children show a striking dissociation between the relatively mild clinical manifestations (tachypnoea, clubbing and chronic cough) and the severity of the pulmonary inflammatory process. Our review describes sJIA-LD as having a reported prevalence of approximately 6.8%, with a mortality rate of between 37% and 68%. It is often associated with an early onset (<2 years of age), macrophage activation syndrome and high interleukin (IL)-18 circulating levels. Other risk factors may be trisomy 21 and a predisposition to adverse reactions to biological drugs. The most popular hypothesis is that the increase in the number of sJIA-LD cases can be attributed to the increased use of IL-1 and IL-6 blockers. Two possible explanations have been proposed, named the “DRESS hypothesis” and the “cytokine plasticity hypothesis”. Lung ultrasounds and the intercellular-adhesion-molecule-5 assay seem to be promising tools for the early diagnosis of sJIA-LD, although high resolution computed tomography remains the gold standard. In this review, we also summarize the treatment options for sJIA-LD, focusing on JAK inhibitors.

## 1. Introduction

Juvenile Idiopathic arthritis (JIA) is the most common rheumatic disease in childhood, with an estimated prevalence of 1 to 4 per 1000 children. Patients with systemic juvenile idiopathic arthritis (sJIA) represent 10–20% of the children with JIA, with incidence rates ranging from 0.4 to 0.8 children per 100,000 children and no gender difference [1]. sJIA can occur during childhood or adolescence but the peak of onset is between 1 and 5 years of age. Although it can occur in patients of any ethnicity, a slightly higher prevalence rate was observed in Japan and India compared to the United States and Canada [2,3]. sJIA is a subtype of JIA with unique clinical manifestations, associated complications and treatment options. sJIA is a diagnosis of exclusion and is defined by the International League of Association for Rheumatology criteria as arthritis of one or more joints, with or preceded by a fever of at least 2 weeks’ duration that is documented to be daily (quotidian) for at least 3 days and accompanied by one or more of the following: (1) evanescent (nonfixed) erythematous rash, (2) generalized lymphadenopathy, (3) hepatomegaly and/or splenomegaly and (4) serositis [4]. Typical symptoms of sJIA are non-specific and could mimic other inflammatory and non-inflammatory diseases [1]. The long-term outcome of sJIA depends on the arthritis evolution while systemic features mostly subside over time [5,6]. Systemic symptoms and the associated complications often require hospitalization for intensive treatment [1]. Macrophage activation disease (MAS) is a secondary macrophage lymphohistiocytosis that complicates approximately 7–17% of patients with sJIA [7]. It is characterized by a cytokine storm, including interferon-γ (IFNγ), increased serum ferritin levels and cytopenia leading progressively to organ failure with an 8–17% mortality rate [8]. Other serious complications in patients with sJIA are myocarditis, arrhythmias, cardiac tamponade and amyloidosis. Recently, more advanced treatments and better control of sJIA has dramatically reduced the incidence of these complications [1]. Pleuritis and pleural effusions were considered the most common pulmonary manifestations of sJIA for decades. Reports of chronic pulmonary disorders during sJIA were exceptionally rare until 2013. Recently, an increase in severe and potentially life-threatening chronic lung disorders (LD) in children with sJIA (sJIA-LD) was reported. These manifestations include pulmonary alveolar proteinosis (PAP), interstitial lung disease (ILD) and pulmonary hypertension (PH) [9].

Pathogenesis of the systemic form of JIA differs from the other subtypes of JIA because it is considered to be predominantly driven by innate rather than by acquired immunity, particularly during the initial phases of the disease. Therefore, sJIA and monogenic autoinflammatory diseases share an altered response of innate immunity to known or yet unknown triggering factors, inducing systemic and/or organ-specific inflammation in predisposed individuals [10]. Arthritis, fever or skin rash are clinical manifestations of some monogenic autoinflammatory diseases as well as of sJIA. Lung involvement is rare, but it can be found in some autoinflammatory diseases, especially in some interferonopathies. STING-associated vasculopathy with onset in infancy (SAVI) is characterized by ILD, fever, malaise, chronic anemia, growth failure, as well as skin involvement [11], making differential diagnosis from sJIA difficult.

### General Aspects of SJIA-LD

sJIA-LD is a unique disease entity characterized by severe chronic pulmonary manifestations and distinctive clinical and immunological features in children with sJIA.

It was originally described to be concomitant with the use of biological drugs anti-interleukin (IL)-1 and anti-IL-6 for sJIA treatment. Kimura et al. [12] firstly observed that 80% of children with SJIA-LD developed pulmonary disease after 2004, following the spread of biological drugs (especially IL-1 inhibitors) for the treatment of sJIA. Similarly, Schulert et al. [9] found that 94% of children with sJIA-LD developed LD after the exposure to at least one biological drug. Therefore, an association of sJIA-LD with the patients’ exposure to biologics acting on IL-1 and IL-6 blockade was firstly postulated by Kimura et al. [12] and then supported by Schulert et al. [9]. 

In 2013, Kimura et al. [12] compared 25 children with sJIA who developed LD (16 patients with PH, 7 with ILD and 5 with PAP) to 389 patients with sJIA enrolled in a pediatric rheumatic disease registry. Patients of the first cohort were significantly more likely to be female, to show systemic features, to be exposed to an IL-1 inhibitor, IL-6 inhibitor (tocilizumab), TNF inhibitor (infliximab), corticosteroids, intravenous immunoglobulin, cyclosporine and cyclophosphamide compared to the patients of the registry cohort. Specifically for the first cohort, MAS was reported during the disease course in 80% of patients and at the diagnosis of lung disease in 60% of cases. At the pulmonary disease onset, 68% of children were taking or recently discontinued a biologic agent, mostly anti-IL1 therapy (48% of patients). The authors found a mortality rate of 68% and the death occurred with a mean of 8.8 months from pulmonary involvement. 

Schulert et al. [9], in a prospective cohort study including 74 children with sJIA enrolled between 2010 and 2018, found lung involvement in 18 children with an sJIA-LD prevalence of 6.8%. The authors observed that patients with sJIA-LD were younger (2 years or younger) at the diagnosis, suffered more frequently from MAS episodes and adverse reactions to biologic therapy compared to children with sJIA without LD. 

Nevertheless, in a recent prospective study including 42 children with newly onset sJIA followed for a median of 5.8 years and in treatment with IL-1 receptor antagonists as a first-line monotherapy, the authors found a lower incidence of LD in sJIA; only one patient with a refractory form of the disease died because of lung involvement with PH and neurological symptoms [13]. 

To better understand the specific relationship between drug reaction and lung disease development, data resulting from the use of anti-cytokine therapy in other diseases could be useful. Indeed, anti-IL-1 and anti-IL-6 drugs are used in other diseases and specifically IL-1 blockers are widely used in the treatment of monogenic autoinflammatory diseases. To date, Saper et al. [14] reported four cases of suspected delayed anakinra reaction in patients with Kawasaki Disease and lung involvement in a patient with an autoinflammatory disease. Therefore, the increasingly widespread use of these drugs could help us to understand the relationship between their use and lung involvement, but to date data are still insufficient and a longer follow-up is needed.

Whether sJIA-LD is associated with the use of biologics or whether its recognition was underestimated in the years preceding the use of such therapies is still unclear. However, given the 6.8% prevalence of LD in children with sJIA [9], it would seem unlikely that sJIA-LD was not recognized earlier.

As aforementioned, several studies confirmed the close association between sJIA-LD and the systemic form of JIA [9,12,15]. Nigrovic et al. [15] described these children as “sickest of the sick”. Despite therapy, 92% of patients still presented with a systemic active disease at the onset of pulmonary involvement [15]. Kimura et al. [12] firstly suggested that the development of sJIA-LD was related to an uncontrolled form of sJIA. 

The underlying mechanism of the lung involvement in sJIA in children is still not clear and continues to be a matter of interest given that not all observations seem to fit a simple causal model. 

## 2. Risk Factors

Several conditions are significantly associated with the onset of sJIA-LD. sJIA-LD mainly occurs in patients who develop sJIA before 2 years of age, who have a history of MAS and high circulating IL-18 levels. Other risk factors include trisomy 21 and a history of adverse reactions to biological drugs, especially tocilizumab. At least 40% of these patients showed adverse reactions to this drug, ranging from malaise to anaphylaxis [9,16,17,18].

## 3. Clinical Features 

In the cohort of 25 patients with sJIA-LD investigated by Kimura et al. [12], the most common clinical symptoms were dyspnea on exertion (72% of cases) and shortness of breath (64%); digital clubbing (40%), cough (44%) and chest pain (20%) were less frequent [9]. The authors also found a devastating clinical evolution: 68% of patients died within a mean of 8.8 ± 11.36 months from lung disease diagnosis [12]. Conversely, Schulert et al. [9] observed a less severe clinical evolution in comparison: stable or improved disease was documented in 14 of 18 patients and a worsening evolution was found in 4 patients, and none died within one year of follow-up.

In a recent multicenter retrospective study including 61 patients, distinctive clinical features were found in subjects with sJIA with lung involvement, such as acute clubbing (61% of cases), atypical rash (56%) and serious adverse reactions to tocilizumab (40%). Pulmonary symptoms were absent in most cases, although hypoxia and PH were present in 43% and 30% of cases, respectively [19]. Other atypical features were eosinophilia (37%) and severe abdominal pain (16%). A noteworthy feature was that the authors reported a higher frequency of digital clubbing compared to Kimura et al. [12], suggesting that this sign could be an early marker of LD [19].

## 4. Pathogenetic Mechanisms 

### 4.1. Pathogenetic Hypotheses

In the literature, the most favored hypothesis is that the increase in sJIA-LD cases can probably be attributed to the use of IL-1 and IL-6 blockers [9,12,19]. Anti-IL1 are only used in systemic JIA while tocilizumab is used in polyarticular forms of refractory JIA in conjunction with treatment with methotrexate and anti-TNF [20]. However, the mechanisms underlying the development of this condition are still unknown. 

Recently, Saper et al. and Chen et al. [19,21] described the characteristics of 61 children with sJIA and chronic parenchymal lung involvement in a retrospective multicenter study. The authors observed that approximately 38% of these patients exposed to tocilizumab showed anaphylactic reactions. A subgroup of these patients exhibited features that fulfilled the criteria for drug reaction with eosinophilia and systemic symptoms (DRESS). The same study group carried out a multicenter case–control study including 66 patients with Still’s disease and features of IL1/IL6-inhibitor-related DRESS and 65 patients with drug-tolerant Still’s disease [14]. They observed that a DRESS-type reaction in the first group of patients was closely correlated with the expression of Human Leukocyte Antigen (HLA) DRB1*15 [14]. Based on these findings, Binstadt et al. [16] proposed two pathogenetic hypotheses. “The DRESS hypothesis” was based on the fact that approximately 50% of patients with sJIA-LD experienced adverse reactions to biological drugs (in particular tocilizumab) fitting the DRESS criteria [22]. The authors proposed that anakinra (interleukin-1 receptor antagonist), canakinumab (IL1β-specific monoclonal antibody) and tocilizumab (or their excipients) led to a pathogenic T cell-response. The drugs could activate T-lymphocytes: (1) by covalently binding to endogenous proteins and being recognized as non-self; (2) by non-covalently binding to the major histocompatibility complex (MHC) and/or T-cell receptor (TCR) outside the antigen-binding pocket, similar to bacterial superantigens [16,23]; (3) by binding within the MHC groove and altering it so that some self-peptides appear as non-self [16]. Thus, IL-1 and IL-6 blockers may achieve one of these interactions with DRB1*15:XX, although anakinra does not share any sequence peptides with canakinumab and tocilizumab. However, these drugs share an excipient, such as polysorbate 80, even if it is a poor candidate trigger for DRESS, being a heterogeneous and almost ubiquitous chemical [16]. Moreover, anakinra should have a very low antigenic potential, being very similar to its endogenous homologue. The reactivation of herpesvirus infections with subsequent activation of CD8+ lymphocytes was observed in many DRESS reactions [16]. However, the underlying mechanism by which DRESS contributes to MAS and/or sJIA-LD is still unknown. It is noteworthy that approximately 25% of patients with sJIA-LD were never exposed to IL-1 or IL-6 blockers, excluding any pathogenic role of biological therapy in this subgroup of patients [16]. The second hypothesis proposed by Binstadt et al. [16] is the “cytokine plasticity hypothesis”, where the biological activity of IL-1 and IL-6 blockers could lead to a favorable condition for adverse reactions similar to drug-induced hypersensitivity syndrome (DIHS) and sJIA-LD in predisposed children [16]. CD4+ cells can change phenotype in response to environmental stimuli. In patients predisposed to develop sJIA-LD, biologic drugs could promote CD4+ cell differentiation into Th1 cells rather than Th17 cells (induced by high levels of IL-1 and IL-6 usually found in sJIA), with a consequent increase in IFNγ production [24].

**Figure 1:**
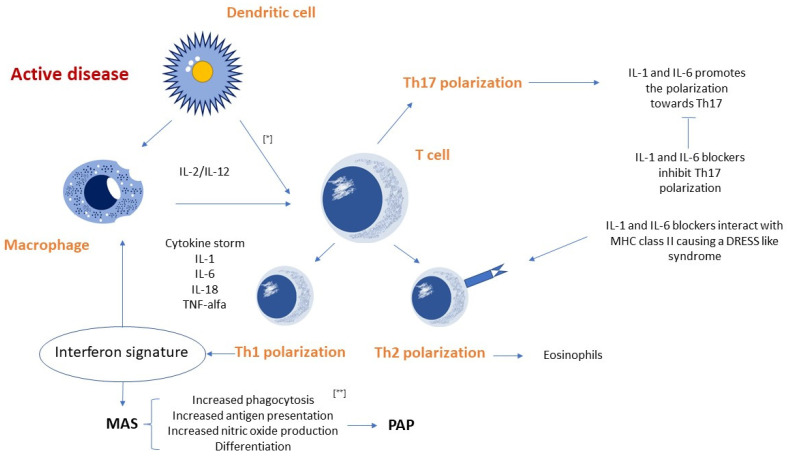
The proposed underlying mechanism of lung involvement in children with systemic juvenile idiopathic arthritis. [*] In cells exposed to interleukin (IL)-2 e IL-12, IL- 18 enhances the synthesis of interferon (IFN) γ, which promotes Th1 differentiation. In particular circumstances, it can trigger a vicious cycle leading to a cytokine storm that favors the development of macrophage activation syndrome. [**] Overexpression of IFN-γ and Th1 cytokines can lead in some murine models to the development of pulmonary alveolar proteinosis.

The proposed underlying mechanism of lung involvement in children sJIA is summarized in Figure 1. 

The Th1 differentiation could be facilitated by other risk factors epidemiologically associated with sJIA-LD, including a young age [16], high levels of IL-18 and other circulating mediators [16,25,26] and trisomy 21 [16,27,28,29]. Patients could develop sJIA-LD without being exposed to anti-IL-1 or anti-IL-6 drugs because that clonal differentiation can occur without exogenous manipulation [16]. Critical issues also underlie the “cytokine plasticity hypothesis”. Indeed, this hypothesis explains the lack of anaphylactic reaction to tocilizumab, although IgE-mediated reactions are not typical of DRESS [30]. It is also unclear how the Th1 transition causes the dysfunction of alveolar macrophages in PAP, which is an unusual pulmonary condition in DRESS [16].

### 4.2. Potential Role of IFNγ in sJIA-LD

Many studies suggested that IFNγ is the main cytokine involved in MAS [25,31,32] probably as part of a positive feedback loop in which lymphocytes activate macrophages. IFNγ may play a key role in the development of sJIA-LD. The activity of IFNγ can be indirectly assessed through the ‘proxy’ molecules induced, such as CXCL9 and CXCL10 [15]. Schulert et al. [9] found higher serum IL-18 levels (27,612 vs. 5413 pg/mL, *p* = 0.047) in sJIA-LD patients compared to patients with sJIA and no lung involvement. IL-18 enhances IFNγ synthesis in cells exposed to cytokines, such as IL-2, IL-12 or IL-15 [33]. Patients with sJIA-LD mostly present a positive history of MAS and high IL-18 levels, indicating an IFNγ overproduction and hence the IFNγ-mediated alveolar macrophage activation [15]. These observations suggest overlapping pathophysiological pathways between MAS and sJIA-LD. Indeed, Schulert et al. [9] found a strong IFNγ-induced gene signature in lung biopsy tissue of patients with sJIA-LD. The authors showed that two of the most upregulated genes were CXCL10 and CXCL9, respectively, increased nine and seven-fold compared to the healthy controls. Furthermore, high concentrations of IL-18, CXCL9 and CXCL10 were found in the bronchoalveolar lavage fluid (BAL) samples of two of the six samples [9]. Consistent with this hypothesis, mice with T-cell-restricted overexpression of T-bet leading to increased IFNγ production showed a dysfunction of bone marrow macrophages, resulting in erythrophagocytosis and dysfunction of alveolar macrophages with the development of a pulmonary disease similar to PAP [34]. These findings confirmed that IFNγ plays a pivotal role in the pathophysiology of sJIA-LD. 

The role of monocytes in sJIA pathogenesis is still unclear, but the monocytes response to IFN may be dysregulated. In an in vitro analysis of monocytes in sJIA, Macaubas et al. [35] found that cells from patients receiving biological therapies showed higher ratios of STAT1 phosphorylation in response to IFNγ than the control cells. In contrast, monocytes from recent onset patients showed lower responses to IFNγ. The authors suggested that anti-cytokine therapy might alter initially defective STAT1 phosphorylation with effects on the IFN signaling pathway, as a higher pSTAT1/IFN response. Similarly, De Jager et al. [36] investigated 16 patients with sJIA treated with anakinra for 3 weeks. They found a clinical improvement and a normalization of IL-1, IL-6 and IL-18 plasma levels, suggesting that anakinra acts as an inflammation regulator blocking IL-1R signaling, but also restores the IL-18-NK cell route.

## 5. Serum Proteome Characteristics in sJIA-LD

In a recent study, Chen et al. [21] investigated the serum proteomes of patients with MAS, sJIA and sJIA-LD. The authors observed that the serum proteomes in active sJIA and MAS are essentially overlapping. The increase in known biomarkers of sJIA, such as amyloid A and S100A9, was reported, as well as the increased levels of heat shock proteins and glycolytic enzymes (e.g., GAPDH, N-acetyl-D-glucosamine and LDH). IL-18 concentrations were increased in all groups (sJIA, particularly in MAS and sJIA-LD). The study of the serum proteome revealed 26 proteins significantly associated with sJIA-LD, with 20 upregulated and 6 downregulated. The most strongly associated was intercellular adhesion molecule 5 (ICAM-5), matrix metalloproteinase 7 (MMP-7), CCL11 (eotaxin 1) and CCL17; while circulating levels of IFNγ-induced chemokines (CXCL9 and CXCL10) were not increased in all patients [21]. Conversely, Schulert et al. [9] found increased levels of IL-18 and IFNγ-induced chemokines CXCL9-10 in the BAL of patients with SJIA-LD. The different sampling method could explain the opposite results of the two study groups: Chen et al. [21] assessed serum concentrations of CXCL9 and CXCL10, while Schulert et al. [9] assessed gene expression levels in lung tissue samples and the concentrations of these proteins in the BAL.

ICAM-5 is the protein most significantly associated with sJIA-LD. MMP-7 is a matrix metallo-peptidase that efficiently cuts ICAM-5, whose levels correlate with ICAM-5 concentration [37]; its levels were also significantly elevated in patients with sJIA-LD. Increased ICAM-5 levels in sJIA-LD patients could be due to the pulmonary activity of this metallo-peptidase [21,38,39].

This study suggested that these two proteins could identify pulmonary disease in the course of sJIA. However, they are not specific to sJIA-LD; they can be increased also in autoimmune/hereditary PAP, idiopathic pulmonary fibrosis, interstitial diseases related to rheumatoid arthritis, pneumonia, PH and in the BAL of children with neuroendocrine hyperplasia, while their levels are normal in patients with sJIA after recovery from lung disease [21,38,40,41,42].

## 6. Diagnostic Imaging

### 6.1. High Resolution Computed Tomography

Lung involvement is common in children with systemic diseases. High-resolution computed tomography (HRCT) is the imaging technique of choice to assess lung involvement in these patients. The aim of HRCT is to detect pulmonary abnormalities, characterize findings, assess the extent of disease and help differential diagnosis. HRCT is also used in the follow-up evaluation and to guide lung biopsy when necessary [43,44,45]. SJIA-LD has unique radiological findings similar to PAP and/or endogenous lipoid pneumonia, often associated with pulmonary vascular changes [19].

In JIA, pleural and pericardial effusions are common findings, affecting about 60% of patients [46,47]. Similar to other connective tissue diseases, lung involvement in JIA commonly manifests firstly as ground glass and evolves towards septal thickening and pulmonary fibrosis, which are the most frequent findings on HRCT in patients with sJIA-LD. In addition, the presence of consolidations (described as a homogeneous increase in pulmonary attenuation that obscures vascular margins) and nodules, progressive lung volume loss and dilation of pulmonary arteries can be observed in patients with sJIA-LD [48].

Lipoid pneumonia is a less common finding in children with sJIA-LD [49,50]. HRCT images show multiple pulmonary nodules, mainly in the centrolobular region, representing intra-alveolar and interstitial cholesterol granulomas resulting from macrophage activation [48]. 

Recently, Vega Fernandez et al. [51] evaluated the pulmonary ultrasonography findings in nine patients with sJIA-LD compared to healthy controls. Seven patients were also eligible for evaluation with HRCT. Consolidation was found in six patients, was always bilateral and occurred in the following distribution: inferior lobe subpleural (six patients), inferior lobe peribronchovascular (six patients), right anterior middle lobe and/or subpleural lingula (four patients), right middle lobe and/or peribronchovascular lingula (three patients), anterior superior lobe subpleural (four patients) and/or superior lobe peribronchovascular (four patients). Less frequent findings included thickening of the interlobular septum (three patients), cysts (two patients) and ground glass opacity (three patients), probably because these are typical abnormalities of the early stages, and these patients were evaluated long after the onset of lung disease. In the study conducted by Saper et al. [19], chest HRCT scans were obtained in 58 of 61 patients, mostly at the time of diagnosis. Most patients had one or more of five patterns. Pattern A was found in 60% of the cases (septal thickening involving the periphery of several lobes, more pronounced in the lower lung areas, parahilary/paramediastinal and/or in the anterior superior lobes with or without adjacent ground-glass opacities). Crazy paving, peribronchovascular consolidation and ground glass opacity were observed in 21%, 22% and 16% of cases, respectively. Contrast-enhanced HRCT scans were performed in 30 of these patients, and 11 subjects (37%) had hyperenhancing lymph nodes [19]. 

Schulert et al. [9] performed chest CT scans in 18 patients with sJIA-LD. They found in sixteen subjects at least one of the seven main lung features and four of them showed at least five of the seven features. Specifically, they observed pleural thickening in 11 patients, septal thickening in 10, bronchial wall thickening or peribronchovascular thickening in 11, tree-in-bud opacity in 2 patients, ground-glass opacity in 8, peripheral consolidation in 5 children and lymphadenopathy in 6. Furthermore, three patients had evidence of pulmonary artery enlargement and one had a diagnosis of PH [9].

### 6.2. Lung Ultrasound 

HRCT is more sensitive at detecting changes in ILD than chest X-rays and represents the gold standard imaging for ILD. However, the significant cumulative radiation and costs associated with serial HRCT preclude its routine use for screening, follow-up and for monitoring responses to therapy [51]. Lung ultrasound (LUS) is a non-invasive, quick, ionizing radiation-free and low-cost imaging technique. Traditionally used for pleural assessment or guided procedures, LUS has recently been proposed for the assessment of ILD in childhood rheumatic diseases. Vega Fernandez et al. [51] found that 106/122 (87%) lung zones assessed using LUS were abnormal, often with severely abnormal subpleural pleural architecture. These findings often spared the axillary zones and affected anterior and posterior fields, as observed in HRCT. Typical LUS abnormalities included a thickened, irregular, fine- and/or coarse-grained hyperechogenic pleural surface with associated scattered or coalesced B-lines, with or without consolidation. In patients with sJIA-LD, focal pleural irregularities were observed in 20 lung fields (16%), diffuse pleural irregularities in 86 fields (70%), B-lines in 106 fields (87%) and subpleural consolidations in 44 (36%) lung fields. Pleural effusion was occasionally found. The authors also showed that focal areas of pleural irregularity associated with scattered B-lines correspond to subpleural reticulation and interlobular septal thickening on HRCT. Scattered, non-confluent B-lines were occasionally observed to be associated with small pleural irregularities < 1 millimeter, and no pleural thickening, granularity, effusions or consolidations were observed in healthy pediatric controls.

In conclusion, acute and chronic pulmonary findings may already be at an advanced stage at the time of clinical onset of lung involvement. Therefore, screening techniques for subclinical disease are required. Indeed, lung function assessment could be useful in patients with JIA to improve patient management and to detect early pulmonary involvement [52]. Moreover, sJIA-LD may have been present since a patient was 2 years of age as children are unable to perform the recommended screening tests for sJIA-LD, including the 6-minute walk test, the lung function tests or an unsedated HRCT. These considerations, together with the costs and exposure to the ionizing radiation of HRCT, suggest a potential role of LUS as a screening tool for sJIA-LD [51].

## 7. Therapy

As in lung diseases, such as asthma [53], and in JIA, the introduction of biological drugs in the last 20 years represented a revolution in the therapeutic management of reducing glucocorticoid (GC) dependency and improving outcomes in children with JIA. The approval of IL-1 and IL-6 blocking therapies in 2012 led to a substantial change in the management of children with sJIA. Additionally, the widespread use of biologic drugs resulted in new insights about the disease mechanisms, with the recognition of a so-called window of opportunity early in the disease course. During this early phase of disease, autoinflammatory mechanisms seem to play a pivotal role, and patients seem to be more responsive to targeted therapy by IL-1 blockade [54]. Based on consensus treatment plans developed by CARRA (Childhood Arthritis and Rheumatology Research Alliance) in the USA, the initial therapy for sJIA most commonly includes four options: starting with GC alone or IL-1 inhibition, IL-6 inhibition or methotrexate, all with or without GC [55].

Patients with SJIA-LD are frequently unresponsive to a single therapy and at least 80% of them require multiple drugs [15].

### 7.1. Anti-Cytokine Therapies

Patients with refractory sJIA are challenging; there is no curative treatment and significant morbidity and a high mortality rate exists. Indeed, a significant number of patients with sJIA show an incomplete response to standard biologic treatments that necessitates sequential trials with multiple biologic and other therapies, and sometimes in combination [22].

#### 7.1.1. IL-1 and IL-6 Blockers

In 2020, Henderson et al. [24] characterized T-cells in patients with acute and chronic sJIA. Patients with acute sJIA had an increased activation of T-regulatory cells with a Th17 gene expression signature; this gene expression signature was found in CD41 T effector cells in patients with chronic arthritis. Therefore, a shift in Th17 response from T-regulatory cells in acute disease to T effector cells in chronic disease occurred [24]. 

Undifferentiated CD4+ cells exposed to IL-1β and/or IL-6 in the presence of TGF-β differentiate into Th17, whereas in the absence of IL-1β and IL-6, these cells become regulatory T cells. However, the latter retain a certain plasticity, in fact, when exposed to IL-1β and IL-6; there is a reduction in the expression of the classic transcription factor of these cells (FOXP3) and an increase in the production of IL-17 [56,57].

Henderson et al. [24] observed higher levels of IL 17+ T-regulatory cells in patients with acute sJIA compared to the controls. In some of these patients, they accounted for 10% of all circulating T-regulatory cells. Furthermore, a significantly lower frequency of circulating IL-17+ regulatory T-cells was observed in patients with new-onset active sJIA treated with anakinra within 4 weeks after diagnosis [24]. Early IL-1 inhibition using anakinra and canakinumab, and IL-6 inhibition using tocilizumab [22] offsets the expression of this Th17 signature and prevents Th17 induction, suggesting the importance of treating within a window of opportunity [54]. sJIA patients treated early with IL-1 blockade often show a rapid and complete therapeutic response, and more than 90% of sJIA patients receiving first-line IL-1 blockade achieved inactivity [13,58].

#### 7.1.2. Etanercept

Etanercept is a fusion protein produced by recombinant DNA. It acts as tumor necrosis factor (TNF) inhibitor, fusing TNF receptor to the constant end of the IgG1 antibody. The American College of Rheumatology response criteria and the Juvenile Arthritis Disease Activity Score based on 10 joints (JADAS-10) established that etanercept is less effective in children with sJIA compared to IL-6 or IL-1 blockers [59,60].

#### 7.1.3. Abatacept

It is a fusion protein composed of the Fc region of the immunoglobulin IgG1 fused to the extracellular domain of CTLA-4. It interferes with the immune activity of T-cells.

The successful use of abatacept in patients with sJIA has been reported in patients with refractory arthritis without systemic features. Apparently, this drug could have a role in restoring immune tolerance, specifically by changing the environment to which T-regulatory cells are exposed in early sJIA [24].

### 7.2. Therapeutic Strategies for Refractory sJIA Associated with Lung Disease 

The advent of Janus kinase (JAK) inhibitor therapy led to new therapeutic options for sJIA. Tofacitinib is a JAK inhibitor, a small molecule targeting JAK1 and JAK3 by blocking the JAK-STAT signaling pathway. The FDA recently approved Tofacitinib for the treatment of polyarticular JIA. Currently, mostly case reports support the use of these drugs in sJIA, MAS and sJIA-LD [61]. The use of JAK/STAT pathway inhibitors in sJIA-MAS and sJIA-LD was extrapolated from animal and clinical studies on similar diseases. Indeed, the beneficial effects of JAK/STAT inhibitors in MAS and HLH were described in mouse models [61,62,63,64]. JAK/STAT inhibition controlled catastrophic hyperinflammation in two MAS mouse models and in a patient with recurrent MAS [65]. A significant clinical improvement and a complete remission within 3 months of starting treatment with tofacitinib were described in a 13-year-old girl with sJIA [66]. In another recent case report, a 4-year-old patient with sJIA-LD was successfully treated with ruxolitinib, a JAK inhibitor with selectivity for JAK1 and JAK2 [63,67]. Other case reports described the use of various therapies in cases of sJIA-LD refractory to classical treatment regimes. In a case report of a 5-year-old boy with progressive sJIA-LD complicated with MAS, the disease was stabilized using several cycles of plasma exchange (PE), after unsuccessful therapy with corticosteroid, methotrexate, cyclosporine, tocilizumab, IVIG and canakinumab. It was suggested that PE was effective because it rapidly decreased circulating cytokine levels, such as IL-18 [68]. Another case report described symptom remission after treatment with canakinumab in a 7-year-old patient with sJIA and PH complicated with a MAS refractory to therapy [69]. Therefore, we can speculate that in refractory forms of sJIA associated with MAS and PH, canakinumab may be a valid therapeutic option. 

In 2022, a case report of a patient with refractory SJIA-LD treated with MAS-825, an investigational bispecific monoclonal antibody targeting IL-1β and IL-18, was reported. Treatment with MAS-825 was associated with a marked reduction in both serum and BAL IL-18 levels with the regression of PAP. After a 10-month treatment with this monoclonal antibody, the patient discontinued treatment with systemic corticosteroids and other biologics dugs without experiencing any side effects [70]. These results suggest that targeting of both IL-1β and IL-18 may be a promising treatment strategy in SJIA-LD. Lastly, a patient with MAS and sJIA-LD who improved with IFNγ antagonist (emapalumab) treatment was also reported in the literature [71]. Thus, this suggests IFNγ could be a target for the treatment of sJIA-LD. 

Regarding non-pharmacologic supportive care, such as chronic home oxygen therapy and positive pressure ventilation, it should be useful in sJIA-LD patients as in patients with ILD. According to the Official American Thoracic Society Clinical Practice Guidelines, home oxygen therapy is recommended in patients with ILD complicated by severe chronic hypoxemia or mild chronic hypoxemia and either dyspnea on exertion or desaturation during sleep. The optimal treatment must be individualized for each patient, given the overlapping nature of these disease states and accounting for previously failed agents, drug interactions, and patient/family preference [72]. 

### 7.3. Therapeutic Strategies for Refractory sJIA Associated with MAS 

To date, no randomized controlled trials about the treatment of sJIA-associated MAS are available. Currently, high-dose GCs (sometimes in association with cyclosporine A), are still the mainstay of treatment [8,73] even though targeted biologic and JAK-inhibition therapies seem to be promising [61].

## 8. Conclusions

Over the last decade, reports of chronic, potentially life-threatening lung manifestations in children with sJIA have dramatically increased. Several theories were proposed to explain this sudden increase in incidence. The most-favored hypothesis associates the appearance of chronic pulmonary manifestations with the common use of biological drugs acting on IL-1 and IL-6 for the treatment of sJIA. However, pulmonary complications can occur in patients never exposed to biological drugs. Despite the enormous progress in understanding this condition, the cause of LD development in some children with sJIA continues to be a critical issue. Clinically, these children show a striking dissociation between the relatively mild clinical manifestations (tachypnoea, clubbing and chronic cough) and the severity of the pulmonary inflammatory process. Therefore, it is important to develop effective methods to stratify sJIA patients with the higher risk of developing lung involvement and strategies for its early diagnosis. We suggest that those children should be tested with a lung function assessment (a six-minute walking test, spirometry and diffusion lung capacity), and in selected cases HRTC should be performed. On one hand, lung function tests require a certain amount of compliance from the patient, who often tends to develop lung involvement at an early age. On the other hand, the execution of HRTC very often requires sedation and exposes the patient to high doses of radiation. To overcome those limitations, lung ultrasound and a serum dosage of specific molecules (i.e., ICAM-5) are safe and could be useful tools. To our knowledge, no specific drug has been approved for the treatment of sJIA-LD. However, JAK inhibitors appear to be the most promising drugs.

## Abbreviations

JIA	Juvenile Idiopathic Arthritis;
sJIA	systemic Juvenile Idiopathic Arthritis;
ILAR	International League of Association Rheumatology;
MAS	Macrophage Activation Syndrome;
LD	Lung Disease;
sJIA-LD	Systemic Juvenile Idiopathic Arthritis with Lung Disease;
PAP	Pulmonary Alveolar Proteinosis;
ILD	Interstitial Lung Disease;
PH	Pulmonary Hypertension;
SAVI	STING-associated vasculopathy with onset in infancy;
IL-1	Interleukin-1;
IL-6	Interleukin-6;
IL-18	Interleukin-18;
DRESS	Drug Reaction with Eosinophilia and Systemic Symptoms;
HLA	Human Leukocyte Antigen;
MHC	Major Histocompatibility Complex;
TCR	T-Cell Receptor;
DIHS	Drug-Induced Hypersensitivity Syndrome;
IFNγ	Interferon gamma;
CXCL9	C-X-C motif chemokine ligand 9;
CXCL10	C-X-C motif chemokine ligand 10;
BAL	Broncho-Alveolar Lavage;
S100A9	S100 calcium-binding protein A9;
GAPDH	Glyceraldehyde 3-Phosphate Dehydrogenase;
LDH	Lactate Dehydrogenase;
ICAM 5	Intercellular Adhesion Molecule 5;
MMP-7	Matrix Metalloproteinase-7;
CCL11	C-C motif chemokine Ligand 11;
CCL17	C-C motif chemokine Ligand 17;
HRCT	High Resolution Computer Tomography;
CT	Computer Tomography;
LUS	Lung Ultrasound;
GC	Glucocorticoid;
CARRA	Childhood Arthritis and Rheumatology Research Alliance;
TGF-β	Transforming Growth Factor beta;
IL-17	Interleukin-17;
FOXP3	Forkhead box P3;
JAK	Janus Kinase;
STAT	Signal Transducer and Activator of Transcription;
Fc region	Fragment Crystallizable region;
JAK1	Janus Kinase 1;
JAK2	Janus Kinase 2.

## References

[1] Lee J.J.Y., Schneider R. (2018). Systemic Juvenile Idiopathic Arthritis. Pediatr. Clin. N. Am..

[2] Fujikawa S., Okuni M. (1997). Clinical analysis of 570 cases with juvenile rheumatoid arthritis: Results of a nationwide retrospective survey in Japan. Pediatr. Int..

[3] Seth V., Kabra S.K., Semwal O.P., Jain Y. (1996). Clinico-immunological profile in juvenile rheumatoid arthritis—An Indian experience. Indian J. Pediatr..

[4] Petty R.E., Southwood T.R., Manners P., Baum J., Glass D.N., Goldenberg J., He X., Maldonado-Cocco J., Orozco-Alcala J., Prieur A.-M. (2004). International League of Associations for Rheumatology classification of juvenile idiopathic arthritis: Second revision, Edmonton, 2001. J. Rheumatol..

[5] Lang B.A., Schneider R., Reilly B.J., Silverman E.D., Laxer R.M. (1995). Radiologic features of systemic onset juvenile rheumatoid arthritis. J. Rheumatol..

[6] Lomater C., Gerloni V., Gattinara M., Mazzotti J., Cimaz R., Fantini F. (2000). Systemic onset juvenile idiopathic arthritis: A retrospective study of 80 consecutive patients followed for 10 years. J. Rheumatol..

[7] Schulert G.S., Grom A.A. (2015). Pathogenesis of Macrophage Activation Syndrome and Potential for Cytokine- Directed Therapies. Annu. Rev. Med..

[8] Minoia F., Davì S., Horne A., Demirkaya E., Bovis F., Li C., Lehmberg K., Weitzman S., Insalaco A., Wouters C. (2014). Clinical Features, Treatment, and Outcome of Macrophage Activation Syndrome Complicating Systemic Juvenile Idiopathic Arthritis: A Multinational, Multicenter Study of 362 Patients: Macrophage Activation Syndrome in Systemic JIA. Arthritis Rheumatol..

[9] Schulert G.S., Yasin S., Carey B., Chalk C., Do T., Schapiro A.H., Husami A., Watts A., Brunner H.I., Huggins J. (2019). Systemic Juvenile Idiopathic Arthritis–Associated Lung Disease: Characterization and Risk Factors. Arthritis Rheumatol..

[10] Lin Y.T., Wang C.T., Gershwin M.E., Chiang B.L. (2011). The pathogenesis of oligoarticular/polyarticular vs. systemic juvenile idiopathic arthritis. Autoimmun. Rev..

[11] Volpi S., Picco P., Caorsi R., Candotti F., Gattorno M. (2016). Type I interferonopathies in pediatric rheumatology. Pediatr. Rheumatol..

[12] Kimura Y., Weiss J.E., Haroldson K.L., Lee T., Punaro M., Oliveira S., Rabinovich E., Riebschleger M., Anton J., Blier P.R. (2013). Pulmonary Hypertension and Other Potentially Fatal Pulmonary Complications in Systemic Juvenile Idiopathic Arthritis: Pulmonary Complications in Systemic JIA. Arthritis Care Res..

[13] Ter Haar N.M., Dijkhuizen E.H.P., Swart J.F., Van Royen-Kerkhof A., El Idrissi A., Leek A.P., De Jager W., De Groot M.C.H., Haitjema S., Holzinger D. (2019). Treatment to Target Using Recombinant Interleukin-1 Receptor Antagonist as First-Line Monotherapy in New-Onset Systemic Juvenile Idiopathic Arthritis: Results From a Five-Year Follow-Up Study. Arthritis Rheumatol..

[14] Saper V.E., Ombrello M.J., Tremoulet A.H., Montero-Martin G., Prahalad S., Canna S., Shimizu C., Deutsch G., Tan S.Y., Remmers E.F. (2022). Severe delayed hypersensitivity reactions to IL-1 and IL-6 inhibitors link to common HLA-DRB1*15 alleles. Ann. Rheum. Dis..

[15] Nigrovic P.A. (2019). Storm Warning: Lung Disease in Systemic Juvenile Idiopathic Arthritis. Arthritis Rheumatol..

[16] Binstadt B.A., Nigrovic P.A. (2022). The Conundrum of Lu.ng Disease and Drug Hypersensitivity-like Reactions in Systemic Juvenile Idiopathic Arthritis. Arthritis Rheumatol..

[17] Weiss E.S., Girard-Guyonvarc’h C., Holzinger D., De Jesus A.A., Tariq Z., Picarsic J., Schiffrin E.J., Foell D., Grom A.A., Ammann S. (2018). Interleukin-18 diagnostically distinguishes and pathogenically promotes human and murine macrophage activation syndrome. Blood.

[18] Maeno N., Takei S., Nomura Y., Imanaka H., Hokonohara M., Miyata K. (2002). Highly elevated serum levels of interleukin-18 in systemic juvenile idiopathic arthritis but not in other juvenile idiopathic arthritis subtypes or in Kawasaki disease: Comment on the article by Kawashima et al. Arthritis Rheum..

[19] Saper V.E., Chen G., Deutsch G.H., Guillerman R.P., Birgmeier J., Jagadeesh K., Canna S., Schulert G., Deterding R., Xu J. (2019). Emergent high fatality lung disease in systemic juvenile arthritis. Ann. Rheum. Dis..

[20] Zaripova L.N., Midgley A., Christmas S.E., Beresford M.W., Baildam E.M., Oldershaw R.A. (2021). Juvenile idiopathic arthritis: From aetiopathogenesis to therapeutic approaches. Pediatr. Rheumatol..

[21] Chen G., Deutsch G.H., Schulert G.S., Zheng H., Jang S., Trapnell B., Lee P.Y., Macaubas C., Ho K., Schneider C. (2022). Identification of Distinct Inflammatory Programs and Biomarkers in Systemic Juvenile Idiopathic Arthritis and Related Lung Disease by Serum Proteome Analysis. Arthritis Rheumatol..

[22] Erkens R., Esteban Y., Towe C., Schulert G., Vastert S. (2021). Pathogenesis and Treatment of Refractory Disease Courses in Systemic Juvenile Idiopathic Arthritis. Rheum. Dis. Clin. N. Am..

[23] Scholl P.R., Diez A., Karr R., Sekaly R.P., Trowsdale J., Geha R.S. (1990). Effect of isotypes and allelic polymorphism on the binding of staphylococcal exotoxins to MHC class II molecules. J. Immunol..

[24] Henderson L.A., Hoyt K.J., Lee P.Y., Rao D.A., Jonsson A.H., Nguyen J.P., Rutherford K., Julé A.M., Charbonnier L.-M., Case S. (2020). Th17 reprogramming of T cells in systemic juvenile idiopathic arthritis. JCI Insight.

[25] Bracaglia C., de Graaf K., Pires Marafon D., Guilhot F., Ferlin W., Prencipe G., Caiello I., Davì S., Schulert G., Ravelli A. (2017). Elevated circulating levels of interferon-γ and interferon-γ-induced chemokines characterise patients with macrophage activation syndrome complicating systemic juvenile idiopathic arthritis. Ann. Rheum. Dis..

[26] Schulert G.S. (2021). The IL-18/IFNγ axis in systemic JIA and MAS—New answers, more questions. Rheumatology.

[27] Sullivan K.D., Lewis H.C., Hill A.A., Pandey A., Jackson L.P., Cabral J.M., Smith K.P., Liggett L.A., Gomez E.B., Galbraith M.D. (2016). Trisomy 21 consistently activates the interferon response. eLife.

[28] Araya P., Waugh K.A., Sullivan K.D., Núñez N.G., Roselli E., Smith K.P., Granrath R.E., Rachubinski A.L., Estrada B.E., Butcher E.T. (2019). Trisomy 21 dysregulates T cell lineages toward an autoimmunity-prone state associated with interferon hyperactivity. Proc. Natl. Acad. Sci. USA.

[29] Sullivan K.D., Evans D., Pandey A., Hraha T.H., Smith K.P., Markham N., Rachubinski A.L., Wolter-Warmerdam K., Hickey F., Espinosa J.M. (2017). Trisomy 21 causes changes in the circulating proteome indicative of chronic autoinflammation. Sci. Rep..

[30] Park E.H., Lee E.Y., Shin K., Kim H.A. (2020). Tocilizumab-induced anaphylaxis in patients with adult-onset Still’s disease and systemic juvenile idiopathic arthritis: A case-based review. Rheumatol. Int..

[31] Avau A., Matthys P. (2015). Therapeutic Potential of Interferon-γ and Its Antagonists in Autoinflammation: Lessons from Murine Models of Systemic Juvenile Idiopathic Arthritis and Macrophage Activation Syndrome. Pharmaceuticals.

[32] Ravelli A., Grom A.A., Behrens E.M., Cron R.Q. (2012). Macrophage activation syndrome as part of systemic juvenile idiopathic arthritis: Diagnosis, genetics, pathophysiology and treatment. Genes Immun..

[33] Prencipe G., Bracaglia C., De Benedetti F. (2019). Interleukin-18 in pediatric rheumatic diseases. Curr. Opin. Rheumatol..

[34] Iriguchi S., Kikuchi N., Kaneko S., Noguchi E., Morishima Y., Matsuyama M., Yoh K., Takahashi S., Nakauchi H., Ishii Y. (2015). T-cell–restricted T-bet overexpression induces aberrant hematopoiesis of myeloid cells and impairs function of macrophages in the lung. Blood.

[35] Macaubas C., Wong E., Zhang Y., Nguyen K.D., Lee J., Milojevic D., Shenoi S., Stevens A.M., Ilowite N., Saper V. (2016). Altered signaling in systemic juvenile idiopathic arthritis monocytes. Clin. Immunol..

[36] de Jager W., Vastert S., Noordman B., Holzinger D., Kuis W., Prakken B., Wulffraat N. (2011). Akinra restores the defective IL-18 NK cell axis in steroid naïve systemic onset JIA patients. Pediatr. Rheumatol..

[37] Conant K., Wang Y., Szklarczyk A., Dudak A., Mattson M.P., Lim S.T. (2010). Matrix metalloproteinase-dependent shedding of intercellular adhesion molecule-5 occurs with long-term potentiation. Neuroscience.

[38] O’Dwyer D.N., Norman K.C., Xia M., Huang Y., Gurczynski S.J., Ashley S.L., White E.S., Flaherty K.R., Martinez F.J., Murray S. (2017). The peripheral blood proteome signature of idiopathic pulmonary fibrosis is distinct from normal and is associated with novel immunological processes. Sci. Rep..

[39] Kennedy B., Branagan P., Moloney F., Haroon M., O’Connell O.J., O’Connor T.M., O’Regan K., Harney S., Henry M.T. (2015). Biomarkers to identify ILD and predict lung function decline in scleroderma lung disease or idiopathic pulmonary fibrosis. Sarcoidosis Vasc. Diffus. Lung Dis. Off. J. Wasog..

[40] Todd J.L., Neely M.L., Overton R., Durham K., Gulati M., Huang H., Roman J., Newby L.K., Flaherty K.R., On behalf of the IPF-PRO Registry Investigators (2019). Peripheral blood proteomic profiling of idiopathic pulmonary fibrosis biomarkers in the multicentre IPF-PRO Registry. Respir. Res..

[41] Wu X., Jeong Y., de Frías S.P., Easthausen I., Hoffman K., Oromendia C., Taheri S., Esposito A.J., Arias L.Q., Ayaub E.A. (2022). Serum proteomic profiling of rheumatoid arthritis–interstitial lung disease with a comparison to idiopathic pulmonary fibrosis. Thorax.

[42] Deterding R.R., Wagner B.D., Harris J.K., DeBoer E.M. (2019). Pulmonary Aptamer Signatures in Children’s Interstitial and Diffuse Lung Disease. Am. J. Respir. Crit. Care Med..

[43] Lynch D.A., Hay T., Newell J.D., Divgi V.D., Fan L.L. (1999). Pediatric diffuse lung disease: Diagnosis and classification using high-resolution CT. Am. J. Roentgenol..

[44] Brody A.S. (2008). New perspectives in imaging interstitial lung disease in children. Pediatr. Radiol..

[45] Klusmann M., Owens C. (2009). HRCT in paediatric diffuse interstitial lung disease—A review for 2009. Pediatr. Radiol..

[46] Society A.T., Society E.R. (2002). American Thoracic Society/European Respiratory Society International Multidisciplinary Consensus Classification of the Idiopathic Interstitial Pneumonias. Am. J. Respir. Crit. Care Med..

[47] Sohn D.I., Laborde H.A., Bellotti M., Seijo L. (2006). Juvenile rheumatoid arthritis and bronchiolitis obliterans organized pneumonia. Clin. Rheumatol..

[48] García-Peña P., Boixadera H., Barber I., Toran N., Lucaya J., Enríquez G. (2011). Thoracic Findings of Systemic Diseases at High-Resolution CT in Children. RadioGraphics.

[49] Schultz R., Mattila J., Gappa M., Verronen P. (2001). Development of progressive pulmonary interstitial and intra-alveolar cholesterol granulomas (PICG) associated with therapy-resistant chronic systemic juvenile arthritis (CJA). Pediatr. Pulmonol..

[50] Athreya B.H., Doughty R.A., Bookspan M., Schumacher H.R., Sewell E.M., Chatten J. (1980). Pulmonary manifestations of juvenile rheumatoid arthritis. A report of eight cases and review. Clin. Chest Med..

[51] Vega-Fernandez P., Ting T.V., Mar D.A., Schapiro A.H., Deluna M.D., Saper V.E., Grom A.A., Schulert G.S., Fairchild R.M. (2022). Lung Ultrasound in Children with Systemic Juvenile Idiopathic Arthritis Associated Interstitial Lung Disease. Arthritis Care Res..

[52] Attanasi M., Lucantoni M., Rapino D., Petrosino M.I., Marsili M., Gasparroni G., Di Filippo P., Di Pillo S., Chiarelli F., Breda L. (2019). Lung function in children with juvenile idiopathic arthritis: A cross-sectional analysis. Pediatr. Pulmonol..

[53] Russo D., Di Filippo P., Attanasi M., Lizzi M., Di Pillo S., Chiarelli F. (2021). Biologic Therapy and Severe Asthma in Children. Biomedicines.

[54] Nigrovic P.A. (2014). Review: Is There a Window of Opportunity for Treatment of Systemic Juvenile Idiopathic Arthritis?: Window of Opportunity in Systemic JIA. Arthritis Rheumatol..

[55] DeWitt E.M., Kimura Y., Beukelman T., Nigrovic P.A., Onel K., Prahalad S., Schneider R., Stoll M.L., Angeles-Han S., Milojevic D. (2012). Consensus treatment plans for new-onset systemic juvenile idiopathic arthritis. Arthritis Care Res..

[56] Zielinski C.E., Mele F., Aschenbrenner D., Jarrossay D., Ronchi F., Gattorno M., Monticelli S., Lanzavecchia A., Sallusto F. (2012). Pathogen-induced human TH17 cells produce IFN-γ or IL-10 and are regulated by IL-1β. Nature.

[57] Li L., Kim J., Boussiotis V.A. (2010). IL-1β–Mediated Signals Preferentially Drive Conversion of Regulatory T Cells but Not Conventional T Cells into IL-17–Producing Cells. J. Immunol..

[58] Nigrovic P.A., Mannion M., Prince F.H.M., Zeft A., Rabinovich C.E., Van Rossum M.A.J., Cortis E., Pardeo M., Miettunen P.M., Janow G. (2011). Anakinra as first-line disease-modifying therapy in systemic juvenile idiopathic arthritis: Report of forty-six patients from an international multicenter series. Arthritis Rheum..

[59] Horneff G., Schulz A.C., Klotsche J., Hospach A., Minden K., Foeldvari I., Trauzeddel R., Ganser G., Weller-Heinemann F., Haas J.P. (2017). Experience with etanercept, tocilizumab and interleukin-1 inhibitors in systemic onset juvenile idiopathic arthritis patients from the BIKER registry. Arthritis Res. Ther..

[60] Russo R.A.G., Katsicas M.M. (2009). Clinical Remission in Patients with Systemic Juvenile Idiopathic Arthritis Treated with Anti-Tumor Necrosis Factor Agents. J. Rheumatol..

[61] Verweyen E.L., Schulert G.S. (2022). Interfering with interferons: Targeting the JAK-STAT pathway in complications of systemic juvenile idiopathic arthritis (SJIA). Rheumatology.

[62] Das R., Guan P., Sprague L., Verbist K., Tedrick P., An Q.A., Cheng C., Kurachi M., Levine R., Wherry E.J. (2016). Janus kinase inhibition lessens inflammation and ameliorates disease in murine models of hemophagocytic lymphohistiocytosis. Blood.

[63] Albeituni S., Verbist K.C., Tedrick P.E., Tillman H., Picarsic J., Bassett R., Nichols K.E. (2019). Mechanisms of action of ruxolitinib in murine models of hemophagocytic lymphohistiocytosis. Blood.

[64] Maschalidi S., Sepulveda F.E., Garrigue A., Fischer A., de Saint Basile G. (2016). Therapeutic effect of JAK1/2 blockade on the manifestations of hemophagocytic lymphohistiocytosis in mice. Blood.

[65] Verweyen E., Holzinger D., Weinhage T., Hinze C., Wittkowski H., Pickkers P., Albeituni S., Verbist K., Nichols K.E., Schulert G. (2020). Synergistic Signaling of TLR and IFNα/β Facilitates Escape of IL-18 Expression from Endotoxin Tolerance. Am. J. Respir. Crit. Care Med..

[66] Huang Z., Lee P.Y., Yao X., Zheng S., Li T. (2019). Tofacitinib Treatment of Refractory Systemic Juvenile Idiopathic Arthritis. Pediatrics.

[67] Bader-Meunier B., Hadchouel A., Berteloot L., Polivka L., Béziat V., Casanova J.-L., Lévy R. (2022). Effectiveness and safety of ruxolitinib for the treatment of refractory systemic idiopathic juvenile arthritis like associated with interstitial lung disease: A case report. Ann. Rheum. Dis..

[68] Sato S., Hosokawa T., Kawashima H. (2022). Successful treatment of plasma exchange for refractory systemic juvenile idiopathic arthritis complicated with macrophage activation syndrome and severe lung disease. Ann. Rheum. Dis..

[69] Paim L.B., Landim M.L.L., Firmino S.L., Monteiro C.M.B.E., Cordeiro L.R., de Araujo F.G., de Carvalho J.F. (2021). Canakinumab for Pulmonary Artery Hypertension and Macrophage Activation Syndrome Associated with Uncontrolled Systemic Juvenile Idiopathic Arthritis. Indian J. Pediatr..

[70] Rood J.E., Rezk A., Pogoriler J., Finn L.S., Burnham J.M., Josephson M.B., Bar-Or A., Behrens E.M., Canna S.W. (2022). Improvement of Refractory Systemic Juvenile Idiopathic Arthritis-Associated Lung Disease with Single-Agent Blockade of IL-1β and IL-18. J. Clin. Immunol..

[71] Lily’s Fight with Macrophage Activation Syndrome: A New Drug Comes to the Rescue. https://systemicjia.org/lilys-fight-with-macrophage-activation-syndrome-mas-new-drug/.

[72] Hayes D., Wilson K.C., Krivchenia K., Hawkins S.M.M., Balfour-Lynn I.M., Gozal D., Panitch H.B., Splaingard M.L., Rhein L.M., Kurland G. (2019). Home Oxygen Therapy for Children. An Official American Thoracic Society Clinical Practice Guideline. Am. J. Respir. Crit. Care Med..

[73] Aytaç S., Batu E.D., Ünal Ş., Bilginer Y., Çetin M., Tuncer M., Gümrük F., Özen S. (2016). Macrophage activation syndrome in children with systemic juvenile idiopathic arthritis and systemic lupus erythematosus. Rheumatol. Int..

